# Improved Anti-Inflammatory Effects of Liposomal Astaxanthin on a Phthalic Anhydride-Induced Atopic Dermatitis Model

**DOI:** 10.3389/fimmu.2020.565285

**Published:** 2020-12-01

**Authors:** Yong Sun Lee, Seong Hee Jeon, Hyeon Joo Ham, Hee Pom Lee, Min Jong Song, Jin Tae Hong

**Affiliations:** ^1^ College of Pharmacy and Medical Research Center, Chungbuk National University, Cheongju, Chungbuk, South Korea; ^2^ Department of Obstetrics and Gynecology, Yeouido St. Mary’s Hospital, College of Medicine, The Catholic University of Korea, Seoul, South Korea

**Keywords:** astaxanthin, liposome, atopic dermatitis, oxidative stress, signal transducer and activator of transcription 3, nuclear factor-κB

## Abstract

Previously, we found that astaxanthin (AST) elicited an anti-inflammatory response in an experimental atopic dermatitis (AD) model. However, the use of AST was limited because of low bioavailability and solubility. We hypothesized that liposome formulation of AST could improve this. In this study, we compared the anti-inflammatory and anti-dermatotic effects of liposomal AST (L-AST) and free AST. We evaluated the effect of L-AST on a phthalic anhydride (PA)-induced animal model of AD by analyzing morphological and histopathological changes. We measured the mRNA levels of AD-related cytokines in skin tissue and immunoglobulin E concentrations in the serum. Oxidative stress and transcriptional activities of signal transducer and activator of transcription 3 (STAT3) and nuclear factor (NF)-κB were analyzed *via* western blotting and enzyme-linked immunosorbent assay. PA-induced dermatitis severity, epidermal thickening, and infiltration of mast cells in skin tissues were ameliorated by L-AST treatment. L-AST suppressed AD-related inflammatory mediators and the inflammation markers, inducible nitric oxide synthase (iNOS) and cyclooxygenase (COX)-2 in PA-induced skin conditions. Oxidative stress and expression of antioxidant proteins, glutathione peroxidase-1 (GPx-1) and heme oxygenase-1 (HO-1), were recovered by L-AST treatment in skin tissues from PA-induced mice. L-AST treatment reduced transcriptional activity of STAT3 and NF-κB in PA-induced skin tissues. Our results indicate that L-AST could be more effective than free AST for AD therapy.

## Introduction

Atopic dermatitis (AD) is a common chronic inflammatory skin disease characterized by mass release of cytokines (1). Stimulated keratinocytes release cytokines and chemokines associated with innate immunity, such as thymic stromal lymphopoietin (TSLP), interleukin (IL)-1β, IL-33, chemokine (C-C motif) ligand (CCL)17, and CCL22 (2). Released mediators have been described to attract macrophages, type 2 T helper cells, and group 2 innate lymphoid cells, which secrete IL-4 and IL-13 to induce immunoglobulin E (IgE) production (3, 4). Mast cells also contribute to AD development and IgE leads to the activation of mast cells through the release of cytokines into skin lesions (5). Thus, the reduction of these cytokines could be an important approach for AD therapy.

The skin is the largest organ of the human body, protecting it from harmful environmental factors, such as chemicals, biologic materials, and allergens. These external materials are known to cause oxidative stress by inducing the generation of reactive species in keratinocytes (6). Inflammatory responses are triggered by oxidative stress through the upregulation of proinflammatory cytokines (7). A previous study showed that TSLP, which is a trigger factor for the pathogenesis of AD, was increased by reactive oxygen species (ROS) in AD tissues (8). A clinical study indicated that the levels of various antioxidants, such as superoxide dismutase (SOD), glutathione peroxidase (GPx), and glutathione (GSH), were substantially decreased in patients with AD compared to healthy controls (9). Thus, increased oxidative stress could contribute to the development of AD.

Signal transducer and activator of transcription 3 (STAT3) and nuclear factor (NF)-κB are critical transcription factors that regulate inflammatory responses; they activate inflammation-related genes, such as cyclooxygenase (COX)-2, tumor necrosis factor (TNF)-α, and IL-6 (10). It is noteworthy that STAT3 is critical for regulating IgE levels and IgE-based allergen sensitization, as well as mast cell degranulation to release cytokines (11, 12). Thus, targeting STAT3 and NF-κB could be a useful approach for AD treatment. The NF-κB inhibitor IMD-0354 alleviated experimental AD in NC/Nga mice through the inhibition of AD-related cytokines and infiltration of inflammatory cells (13). Topical NF-κB decoy oligonucleotides were shown to block chronic AD-like skin inflammation by the downregulation of Th1 and Th2 cytokines (14). Topical application of Momelotinib, a novel Janus kinase (JAK)1/JAK2 inhibitor, suppressed STAT3 signaling, the release of pro-inflammatory cytokines, total serum IgE levels, and mast cell infiltration, and thus improved the symptoms of AD (15).

Astaxanthin (3,3’-dihydroxy-β,β-carotene-4,4’-dione; AST) is a xanthophyll carotenoid usually found in microalgae and crustaceans, such as krill and shrimp (16). Previous studies have shown that AST is pharmacologically effective against various diseases, including cardiovascular, gastrointestinal, liver, neurodegenerative, and skin diseases, *via* its anti-oxidant and anti-inflammatory activities (17, 18). Our previous reports revealed that AST inhibited oxidative stress and inflammation *via* inactivation of STAT3 and NF-κB in ethanol-induced liver injury and lipopolysaccharide-induced neuroinflammation (19, 20). Topical application has been shown to provide advantages by reducing side effects, drug abuse and toxicity, allowing for high-dose application, while being easy to use, and avoiding first-pass metabolism (21, 22). Moreover, the topical route is well-suited for sustained and controlled delivery over a prolonged period (21). With respect to skin diseases, topical application directly at the site of skin inflammation is indicated. However, astaxanthin has poor water-solubility, thus limiting direct application to the skin (23, 24). To solve this problem, liposomal formulation improves its solubility by conjugation with phospholipid structures (25).

In this study, we investigated the effect of liposomal AST (L-AST) on the prevention of AD *via* inhibition of skin inflammation.

## Materials and Methods

### Preparation of Liposomal Astaxanthin

AST, purchased from GDE Co., Ltd. (Siheung, Korea), was mixed with 70% ethanol at room temperature (21–25°C), and insoluble materials were removed using Whatman filter paper. Phosphatidylcholine (from Soy) was dissolved in 95% ethanol, and insoluble materials were removed using Whatman filter paper. AST and phosphatidylcholine were mixed at a 1:4 ratio using a high-pressure homogenizer (Microfluidizer™, cooling temperature: −15~–20°C/1,000 bar/3 cycles; 11-6094A000, Microfluidics Corp., MA, USA). After mixing, ethanol was removed *via* vacuum distillation. The solution was transferred to a 5 mm stainless steel plate after which all liquid components were removed using a vacuum freeze dryer. The solids from which the liquid components had been removed were collected and stored at room temperature to prepare L-AST for use in the experiment. The particle size of the L-AST produced was on average 64.5 ± 2.8 nm, which was determined using a particle size analyzer (ELS-Z, Otsuka electronics, Osaka, Japan).

### Animal Housing and Ethical Approval

Animal experiments were performed in accordance with the guidelines for animal experiments of the Institutional Animal Care and Use Committee (IACUC) of the Laboratory Animal Research Center at Chungbuk National University, Korea. The experimental protocol was approved by the IACUC of the Laboratory Animal Research Center at Chungbuk National University, Korea (Ethical approval No. CBNUA-1304-19-01). Male SKH-1 mice (also known as HR-1 hairless mice) were obtained from Daehan Bio Link Co., Ltd. (Eumsung, Korea) and housed at controlled temperature (21–25°C), relative humidity (55 ± 10%), and 12 h light-dark cycles. Food and purified tap water were provided *ad libitum*.

### Phthalic Anhydride (PA)-Induced AD Development

SKH-1 mice (8-week-old males) were randomly divided to five groups. PA solution (5%, 200 μl) was applied on the dorsal skin three times a week for 4 weeks. AST (1 mg/ml; 200 μl) or L-AST (0.5 and 1 mg/ml; 200 μl) was applied 3 h after every PA treatment. Vehicle-treated mice were used as a control. PA, AST, and L-AST were dissolved in acetone:olive oil (AOO) solution (4:1 ratio, v/v). The clinical score for each group was determined as none (0), mild (1), moderate (2), or severe (3) according to the average score of each symptom, i.e., erythema (redness), scaling, and itching, for each mouse.

### Measurement of Body and Lymph Node Weight

During the experimental period, the body weight of mice was measured once a week over the course of 4 weeks using an electronic scale (Mettler Toledo, Greifensee, Switzerland). Skin draining lymph nodes were collected and weighed using a precision electronic balance (FX-200i; A&D Korea, Seoul, Korea).

### Immunohistochemistry (IHC)

IHC was conducted as described previously (26). The slides were stained with specific primary antibodies. Mast cells were stained using toluidine blue solution (IHC world, Ellicott City, MD, USA). The average of epidermal thickness and the number of mast cells was calculated by a single measurement of six different fields (non-overlapping) in each group. Information on the antibodies used is provided in **Supplementary Table S1**.

### Western Blot Analysis

Western blot analysis was performed as previously described (26). The membrane was incubated with specific primary antibodies directed against the following proteins: p50, GPx-1, heme oxygenase (HO)-1, STAT3, histone H1 and β-actin (Santa Cruz Biotechnology, CA, USA), p65, inducible nitric oxide synthase (iNOS) and COX-2 (Abcam, Cambridge, MA, USA), and p-IκBα, IκBα and p-STAT3 (Cell Signaling, Beverly, MA, USA). Histone H1 and β-actin were used as loading controls. Band intensities were measured using the Fusion FX7 image acquisition system (Vilber Lourmat, Eberhardzell, Germany). Western blot band intensities were quantified using ImageJ software (NIH; Bethesda, MD, USA). Information on the antibodies used is provided in **Supplementary Table S1**.

### Quantitative Real-Time PCR (RT-qPCR)

RT-qPCR was performed as described previously (26). Briefly, total RNA was collected from mouse skin tissues or lymph nodes using the Ribo^EX^ RNA Extraction Kit (GeneAll Biotechnology, Seoul, Korea) and cDNA was synthesized using the High-Capacity RNA-to-cDNA kit (Applied Biosystems, Foster City, CA, USA). RT-qPCR was performed using specific primers with the StepOnePlus™ PCR System (Applied Biosystems, Foster City, CA, USA). Levels of mRNA were normalized to the 18S sequence, which was used as a house-keeping control. The fold change between groups was determined for all targets using the 2^ΔΔCt^ method. Specific primer sequences are described in **Supplementary Table S2**.

### Serum IgE Assay

Blood was collected from sacrificed mice and serum was isolated *via* centrifugation (3,500 rpm for 7 min at 4°C) using blood collection tube (BD Microtainer^®^, Franklin Lakes, NJ, USA). Serum levels of mouse IgE were measured using the ELISA kit of KOMA Biotech (Seoul, Korea) according to the manufacturer’s protocol.

### Oxidative Stress Assay

Hydrogen peroxide (H_2_O_2_) was measured using the Hydrogen Peroxide Assay Kit (Biovision, Milpitas, CA, USA). The levels of GSH and oxidized glutathione (GSSG) were analyzed using the GSH/GSSG Ratio Detection Assay Kit (Abcam, Cambridge, MA, USA). Malondialdehyde (MDA) levels were measured using TBARS Assay Kit according to the manufacturer’s instructions (Cayman, Ann Arbor, MI, USA).

### Statistical Analysis

All experiments were repeated at least three times to ensure reproducibility of the results. Statistical analysis was performed with GraphPad Prism 4.03 software (San Diego, CA, USA). Group differences were analyzed by one-way analysis of variance followed by Tukey’s multiple comparison test. All values are presented as the mean ± SD. Significance was set at a p < 0.05 for all tests. *Control *vs.* PA; ^†^PA *vs.* PA + AST1, PA + L-AST0.5, and PA + L-AST1; ^‡^PA + AST1 *vs.* PA + L-AST0.5 and PA + L-AST1.

## Results

### Effects of L-AST Treatment on Experimental AD Development

**Figure 1:**
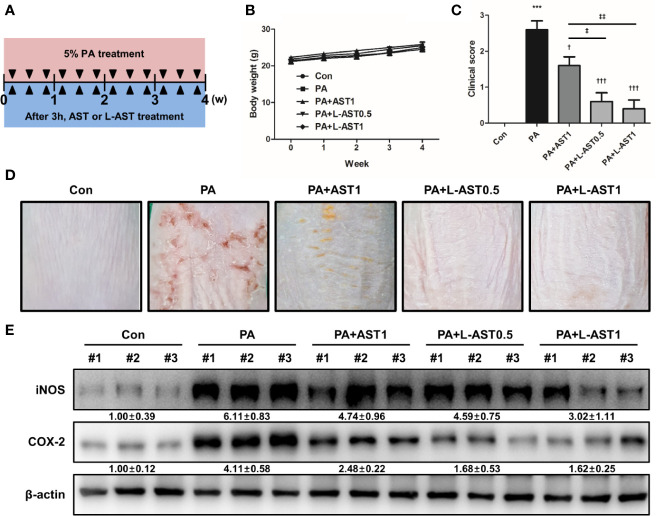
Liposomal astaxanthin inhibits PA-induced atopic dermatitis. **(A)** Experimental schedule of PA-induced atopic dermatitis model. Mice were treated with 5% PA solution three times a week for 4 weeks. AST or L-AST were applied 3 h after every PA treatment. **(B)** Body weight changes during 4-week experiment. **(C, D)** The morphological changes in mice after 4-week treatment as described in the *Materials and Methods*. Bar graphs indicate clinical score **(C)**. *n* = 6. The photographs are representative of each group of mice **(D)**. **(E)** Expression of inflammation markers, COX-2 and iNOS, in skin tissues of each group. #1–3 refer to skin tissues of the different mice in the same group. ^†,‡^ *p* < 0.05, ^‡‡^ *p* < 0.01 and ***,^†††^ *p* < 0.001.

PA exposure is known to cause irritation, potentially leading to allergic skin diseases, such as contact dermatitis and AD by inducing allergy-related cytokines, chemokines, and IgE (27, 28). To investigate whether L-AST had an improved potential to prevent AD compared to AST, we used a PA-induced AD model. As shown in **Figure 1A**, we induced experimental AD. First, we measured the changes in body weight over the course of the experimental period. No substantial body weight changes were detected upon either AST or L-AST treatment (**Figure 1B**). Next, we compared the associated AD symptoms, consisting of erythema (redness), scaling, and itching, for each group. It was revealed that AD symptoms and clinical scores were increased in the PA-induced group (**Figures 1C, D**). However, these symptoms and scores were significantly reduced upon AST treatment (**Figures 1C, D**). L-AST treatment further reduced PA-induced AD development in mice when compared with AST-treated mice (**Figures 1C, D**). As shown in **Figure 1D**, AST dyed the skin surface red, whereas L-AST did not color the skin. Western blot analysis showed that the reduction in the expression of the inflammation markers COX-2 and iNOS was more pronounced in L-AST treated mice when compared to AST treated mice (**Figure 1E**).

### Effect of L-AST Treatment on AD-Like Skin Inflammation

**Figure 2:**
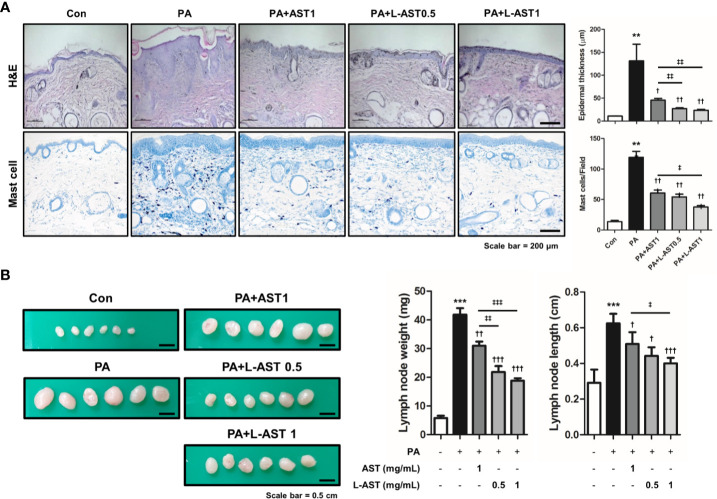
Liposomal astaxanthin reduces epidermal thickening, lymph node weight, and expression of inflammatory markers. **(A)** Histological changes in mice after 4-week PA treatment. Bar graphs indicate epidermal thickness and mast cell number. *n = 6*. **(B)** Changes of skin draining lymph node weight and length (major axis) of each group. *n* = 6. ^†,‡^ *p* < 0.05, ^**,††^,^‡‡^ *p* < 0.01, and ^***^,^†††,‡‡‡^ *p* < 0.001.

**Figure 3:**
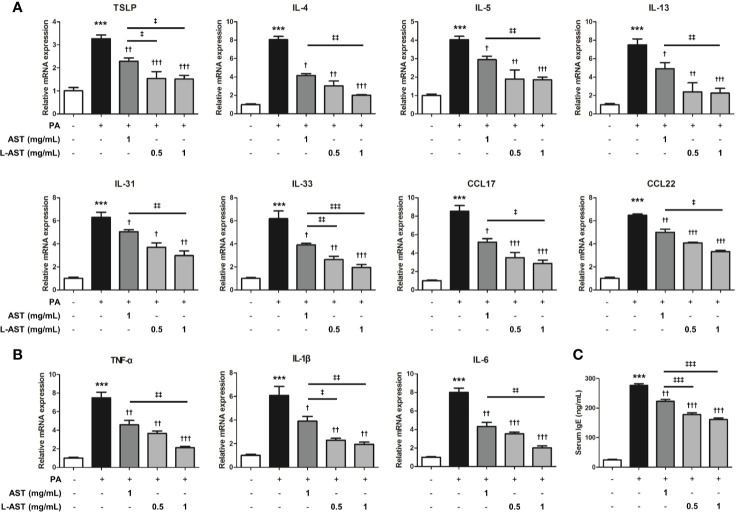
Liposomal astaxanthin suppresses skin inflammation. **(A)** mRNA expression of Th2-related cytokines, TSLP, IL-4, IL-5, IL-13, IL-31, IL-33, CCL17, and CCL22 in skin tissues. *n* = 4. **(B)** mRNA expression of Th1-related cytokines, TNF-α, IL-1β, and IL-6, in skin tissues. *n* = 4. **(C)** The serum concentration of IgE. *n* = 6. ^†,‡^ *p *< 0.05, ^**††,‡‡^ *p* < 0.01, and ^***^,^†††,‡‡‡^ *p* < 0.001.

Histological analysis of the skin tissues showed that AST and L-AST treatment reduced epidermal thickening in PA-induced skin tissues, with L-AST being more effective than free AST (**Figure 2A**). The reduction in PA-induced mast cell infiltration was also more pronounced in L-AST-treated mice than in AST-treated mice (**Figure 2A**). We further investigated the weight of skin draining lymph nodes, which serve as an indicator of skin inflammation. The result showed that AST and L-AST moderately and sufficiently reduced the PA-induced increase in lymph node weight and length (major axis), respectively (**Figure 2B**). Various inflammatory cytokines and chemokines are critical factors for AD development (2). Therefore, we analyzed AD-related mediators of PA-induced skin conditions. RT-qPCR analysis showed that the expression of Th2-related cytokines and chemokines, including TSLP, IL-4, IL-5, IL-13, IL-31, IL-33, CCL17, and CCL22, was increased in PA-induced skin tissues, but treatment with AST reduced the expression of these cytokines (**Figure 3A**). Furthermore, L-AST suppressed Th2-related mediators more effectively (**Figure 3A**). The increase in PA-induced Th1 cytokines, such as TNF-α, IL-1β, and IL-6, was markedly reduced in skin tissues of L-AST-treated mice compared to AST-treated mice (**Figure 3B**). Next, we evaluated serum levels of IgE, an indicator of allergic inflammation. Serum IgE was increased in PA-treated mice; these PA-induced serum IgE levels were significantly reduced in AST-treated mice (**Figure 3C**). L-AST was more efficient in decreasing serum IgE levels than free AST (**Figure 3C**). Further analysis indicated that L-AST treatment reduced AD-related cytokine levels (IL-1β, IL-6, IL-4, and IL-13) in lymph nodes to a greater extent than AST treatment (**Supplementary Figure S1**).

### Effect of L-AST Treatment on Oxidative Stress

**Figure 4:**
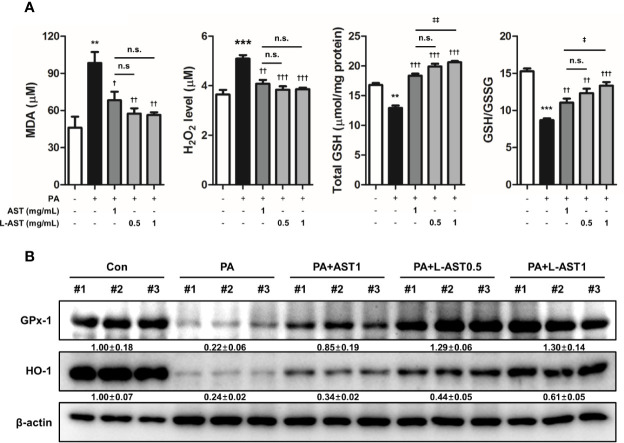
Liposomal astaxanthin alleviates oxidative stress in PA-induced skin tissues. **(A)** MDA levels, H_2_O_2_ levels, total GSH, and GSH/GSSG ratio in skin tissues. *n* = 6. **(B)** Expression of antioxidant-related markers, GPx-1, and HO-1, in skin tissues. #1–3 refer to skin tissues of the different mice in the same group. ^†,‡^ *p* < 0.05, ^**,††,‡‡^ *p *< 0.01, and ^***,†††,‡‡‡^ *p* < 0.001.

Previous studies have shown that oxidative stress is related to AD (7, 9). Furthermore, AST possesses antioxidant properties (29). Therefore, we evaluated oxidative stress in PA-induced skin conditions. The level of MDA, a marker of lipid peroxidation, was elevated by PA treatment, but reduced by the application of free AST and L-AST (**Figure 4A**). Additionally, PA-induced H_2_O_2_ levels were alleviated by free AST and L-AST treatments (**Figure 4A**). In addition, decreased GSH levels and GSH/GSSG ratios were recovered by the treatment of AST and L-AST (**Figure 4A**). Treatment with the latter resulted in enhanced suppression of oxidative stress compared to AST treatment (**Figure 4A**). We further investigated the expression of antioxidant-related genes, such as GPx-1 and HO-1. Western blot analysis showed that reduced expression of HO-1 and GPx-1 was recovered by AST in PA-induced mice (**Figure 4B**). L-AST was more effective in reversing GPx-1 and HO-1 expression levels compared to free AST (**Figure 4B**).

### Effect of L-AST Treatment on STAT3 and NF-κB Signaling Activation

**Figure 5:**
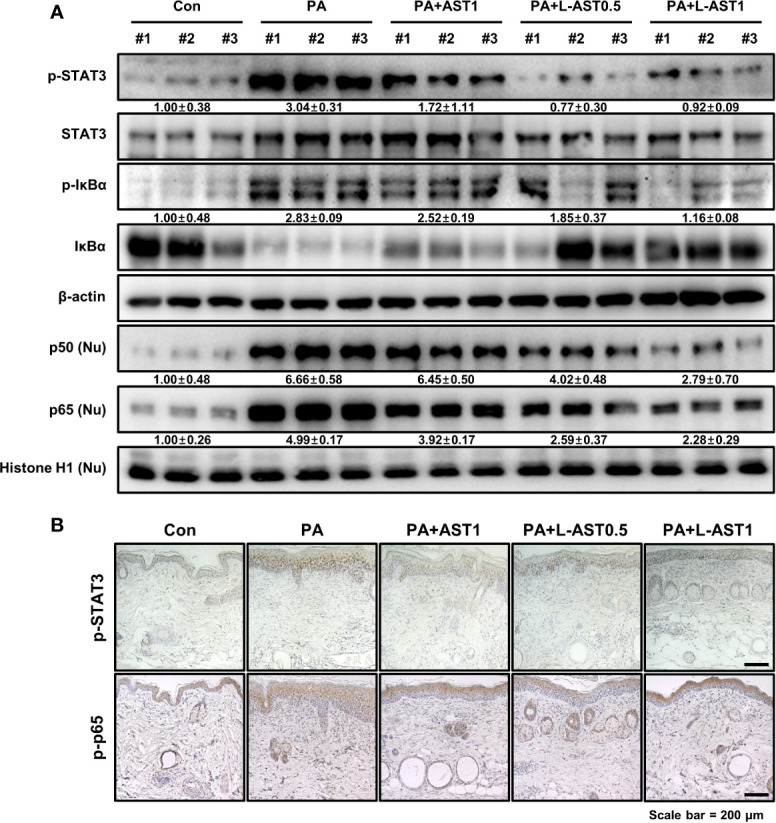
Effect of liposomal astaxanthin on STAT3 and NF-κB signaling in PA-induced AD model. **(A)** Expression of phosphorylated STAT3 and IκBα in whole tissue lysates and nuclear translocation of p50 and p65 in nuclear fractions by Western blot analysis in skin tissues. #1–3 refer to skin tissues of the different mice in the same group. **(B)** Representative IHC images showing phospho-STAT3 and phospho-p65 in PA-induced skin tissues.

STAT3 and NF-κB are both involved in various inflammatory diseases including AD. Recent studies demonstrated that ROS induced the activation of STAT3 and NF-κB signaling (30, 31). To investigate whether L-AST treatment further inhibited STAT3 and NF-κB activation in a PA-induced AD model, we analyzed the activation of STAT3 and NF-κB using western blot and IHC analysis. PA-treated mice were characterized by an increase in the phosphorylation of IκBα and STAT3 in cytoplasmic fractions, and by nuclear translocation of p50 and p65, as revealed by comparing cytoplasmic and nuclear fractions (**Figure 5A**). In contrast, AST treatment inhibited STAT3 and NF-κB activation (**Figure 5A**). In addition, L-AST treatment suppressed the activation of STAT3 and NF-κB more efficiently than free AST (**Figure 5A**). Similar to western blot analysis, IHC showed that AST treatment reduced phosphorylation of STAT3 and p65 in skin tissues upon PA treatment (**Figure 5B**). Moreover, L-AST was more potent than AST in inhibiting the activation of STAT3 and p65 in PA-induced skin conditions.

## Discussion

AD is a chronic inflammatory skin disease, which is characterized by increased IgE levels and inflammatory cytokine expression (32, 33). Targeting of AD-related inflammatory mediators and anti-inflammatory compounds are promising approaches for AD therapy (34). Previous studies reported that green tea extracts from tannase digests inhibited skin inflammation and mast cell infiltration in house dust mite antigen-induced AD-like lesions (35). Ethanol extracts of *Ampelopsis brevipedunculata* rhizomes inhibited an AD-like skin inflammation through downregulation of serum IgE levels and expression of TNF-α, interferon (IFN)-γ, IL-4, IL-13, and IL-31 in a BALB/c AD model (36). In a previous study, we also found that *Centella asiatica* reduced PA-induced AD through its anti-inflammatory effects (37). In line with these findings, in our present study, significantly reduced AD-related cytokine release was observed in the L-AST-treated group. These data indicate that L-AST constitutes a promising candidate for the development of a therapeutic agent for AD treatment.

Oxidative stress has been associated with inflammatory diseases (30). Accordingly, endogenous and environmental pro-oxidants could induce ROS, causing oxidative damage, such as DNA modifications, lipid peroxidation, and inflammatory responses (38). However, the relationship between oxidative stress and AD is not clear. Previous reports were in support of suppression of oxidative stress being capable of alleviating AD. N-acetyl-L-cysteine, a precursor of GSH, decreased the levels of IL-4, IL-5, and IFN-γ in Th2 cells (39). A clinical study showed that MDA levels were increased, but antioxidant parameters, including SOD, GSH, GPx, and vitamins (A,C, and E), were significantly decreased in blood samples from patients with AD compared to healthy controls (9). It has been reported that urinary concentrations of pentosidine and 8-hydroxy-2’-deoxyguanosine were higher in patients with AD compared to the controls (40). Furthermore, our data revealed that treatment with free AST or L-AST could decrease the levels of MDA and H_2_O_2_, but resulted in an increased GSH/GSSG ratio. These factors implicated that inhibition of oxidative stress may contribute to preventing AD.

STAT3 and NF-κB are transcriptional factors significantly contributing to the development of AD as they play important roles in the regulation of AD-related inflammatory mediators (14, 41). Several STAT3 inhibitors, such as momelotinib, a novel JAK1/JAK2 inhibitor, and the JAK inhibitor JTE-052 downregulated the STAT3-specific signal, release of pro-inflammatory cytokines, total serum IgE levels, and mast cell numbers, and concomitantly improved the symptoms of AD (15, 42). NF-κB inhibitors alleviated AD-like skin inflammation by inhibiting of inflammatory regulators and the infiltration of immune cells (13, 14). Our results showed that L-AST inhibited phosphorylation of IκBα and STAT3, as well as nuclear translocation of p50 and p65 in PA-induced skin conditions. These data suggest that L-AST-mediated inhibition of STAT3 and NF-κB could be essential for its anti-AD effect.

Topical administration has several advantages for treatment of skin diseases. Transdermal delivery can avoid metabolism of drugs by the liver, reduce side effects, and achieve local effects (43). Topical administration of AST effectively inhibited UV-induced ocular photokeratitis and AD-like skin inflammation (44, 45). Nevertheless, AST is both hydrophobic and hydrophilic and has poor water-solubility, rendering it unsuitable for skin application. To overcome this problem, various methods have been developed (46). Among them, liposome formulation is widely used for skin delivery systems as it is associated with increased drug solubilization (46). Additionally, liposome formulation could increase the transdermal delivery of AST. As demonstrated by many studies, liposome formulations are superior to the free forms with regard to skin delivery (47). A previous study indicated that liposomal adenosylcobalamin hydrogel improved skin permeation and reduced AD symptoms more efficiently than the non-liposomal type in a dichloronitrobenzene-induced AD mouse model (48). It has also been reported that liposomal betamethasone exerted stronger increased anti-inflammatory actions in patients with AD than in controls (49). It needs to be confirmed in future studies whether L-AST has increased skin permeability compared to AST.

This study demonstrated that L-AST treatment could prevent inflammatory cytokine release and oxidative stress in a PA-induced AD model more efficiently than free AST. Thus, liposomal formulation enhances the therapeutic efficacy of AST and has more practical applicability.

## Data Availability Statement

The original contributions presented in the study are included in the article/**Supplementary Material**. Further inquiries can be directed to the corresponding authors.

## Ethics Statement

The animal study was reviewed and approved by Institutional Animal Care and Use Committee (IACUC) of Laboratory Animal Research Center at Chungbuk National University, Korea (Ethical approval No. CBNUA-1304-19-01).

## Author Contributions

YL conducted most of the experiments, performed data analyses, designed the experiments, and wrote the manuscript. SJ and HH assisted in animal experiments. HL provided experimental advices. MS and JH supervised the entire project and contributed profoundly to experimental design, data interpretation, and revision the manuscript. All authors contributed to the article and approved the submitted version.

## Funding

This work is financially supported by the National Research Foundation of Korea [NRF] grant funded by the Korean government (MSIP) (No. MRC, 2017R1A5A2015541).

## Conflict of Interest

The authors declare that the research was conducted in the absence of any commercial or financial relationships that could be construed as a potential conflict of interest.
