# Supplementary material for: Improved Anti-Inflammatory Effects of Liposomal Astaxanthin on a Phthalic Anhydride-Induced Atopic Dermatitis Model
Source: Front Immunol. 2020 Dec 1;11:565285. doi: 10.3389/fimmu.2020.565285 (PMC7736086; doi:10.3389/fimmu.2020.565285)
Supplement: Supplementary file 1 [file DataSheet_1.docx]

**<Supplementary Information>**

**Improved anti-inflammatory effects of liposomal astaxanthin on phthalic anhydride-induced atopic dermatitis model**

Yong Sun Lee^1^, Seong Hee Jeon^1^, Hyeon Joo Ham^1^, Hee Pom Lee^1^, Min Jong Song^2*^ and Jin Tae Hong^1*^

^1^College of Pharmacy and Medical Research Center, Chungbuk National University, 194-31 Osongsaengmyeong 1-ro, Osong-eup, Heungduk-gu, Cheongju, Chungbuk, 28160, Republic of Korea

^2^Department of Obstetrics and Gynecology, Yeouido St. Mary’s Hospital, College of Medicine, The Catholic University of Korea, 10, 63-ro, Yeongdeungpo-gu, Seoul, 07345, Republic of Korea

^*^**Corresponding authors:**

Jin Tae Hong, Ph. D., College of Pharmacy and Medical Research Center, Chungbuk National University, 194-31 Osongsaengmyeong 1-ro, Osong-eup, Heungduk-gu, Cheongju, Chungbuk, 28160, Republic of Korea

Tel.: 82-43-261-2813, Fax: 82-43-268-2732, E-mail: jinthong@chungbuk.ac.kr

Min Jong Song, M.D., Ph.D., Department of Obstetrics and Gynecology, Yeouido St. Mary’s Hospital, College of Medicine, The Catholic University of Korea, 10, 63-ro, Yeongdeungpo-gu, Seoul, 07345, Republic of Korea

Tel.: 82-2-3779-1069, E-mail: bitsugar@catholic.ac.kr

**Supplementary Table S1. Antibody and kit information**

| **Name (Clone)** | **Cat. No.** | **Application** | **Supplier** |
| --- | --- | --- | --- |
| **Primary antibody** | | | |
| **iNOS** | **ab15323** | **WB** | **Abcam** |
| **COX-2 (EPR12012)** | **ab179800** | **WB** | **Abcam** |
| **p65** | **ab16502** | **WB** | **Abcam** |
| **Phospho-p65** | **ab86299** | **IHC-P** | **Abcam** |
| **Phospho-IκBα** | **5209** | **WB** | **Cell Signaling** |
| **IκBα** | **4814** | **WB** | **Cell Signaling** |
| **Phospho-STAT3 (D3A7)** | **9145** | **WB** | **Cell Signaling** |
| **p50 (NLS)** | **sc-114** | **WB** | **Santa Cruz Bio** |
| **GPx-1 (N-20)** | **sc-22146** | **WB** | **Santa Cruz Bio** |
| **HO-1 (H-105)** | **sc-10789** | **WB** | **Santa Cruz Bio** |
| **STAT3 (F-2)** | **sc-8019** | **WB** | **Santa Cruz Bio** |
| **β-actin (C4)** | **sc-47778** | **WB** | **Santa Cruz Bio** |
| **Histone H1 (H-2)** | **sc-393358** | **WB** | **Santa Cruz Bio** |
| **Secondary antibody** | | | |
| **Anti-mouse IgG, HRP-linked** | **7076** | **WB** | **Cell Signaling** |
| **Anti-rabbit IgG, HRP-linked** | **7074** | **WB** | **Cell Signaling** |
| **Anti-goat IgG H&L** | **ab6741** | **WB** | **Abcam** |
| **Kit** | | | |
| **NovaUltra Toluidine Blue Stain Kit** | **IW-3013** | **IHC-P** | **IHC World** |
| **Mouse IgE ELISA Kit** | **K3231082** | **ELISA** | **KOMA Biotech** |
| **Hydrogen Peroxide Assay Kit** | **K265** |  | **BioVision** |
| **GSH/GSSG Ratio Detection Assay Kit** | **ab138881** |  | **Abcam** |
| **TBARS Assay Kit** | **10009055** |  | **Cayman** |

**Supplementary Table S2. Primer sequence information**

| **Primer** | **Species** | **Sequence (Forward)** | **Sequence (Reverse)** |
| --- | --- | --- | --- |
| **18S** |  | **AGGAATTGACGGAAGGGCACCA** | **GTGCAGCCCCGGACATCTAAG** |
| **IL-4** | **mouse** | **GGTCTCAACCCCCAGCTAGT** | **GCCGATGATCTCTCTCAAGTGAT** |
| **IL-5** | **mouse** | **CACCGAGCTCTGTTGACAAGC** | **GAGTAGGGACAGGAAGCCTCATC** |
| **IL-13** | **mouse** | **CCTGGCTCTTGCTTGCCTT** | **GGTCTTGTGTGATGTTGCTCA** |
| **IL-31** | **mouse** | **CACACAGGAACAACGAAGCC** | **CGATATTGGGGCACCGAAG** |
| **IL-33** | **mouse** | **TCCAACTCCAAGATTTCCCCG** | **CATGCAGTAGACATGGCAGAA** |
| **CCL17** | **mouse** | **GACGACAGAAGGGTACGGC** | **GCATCTGAAGTGACCTCATGGTA** |
| **CCL22** | **mouse** | **AGGTCCCTATGGTGCCAATGT** | **CGGCAGGATTTTGAGGTCCA** |
| **TNF-α** | **mouse** | **CACTTGGTGGTTTGCTACGA** | **TCTTCTCATTCCTGCTTGTGG** |
| **IL-1β** | **mouse** | **CCTTCCAGGATGAGGACATGA** | **TGAGTCACAGAGGATGGGCTC** |
| **IL-6** | **mouse** | **GAGGATACCACTCCCAACAGACC** | **AAGTGCATCATCGTTGTTCATACA** |


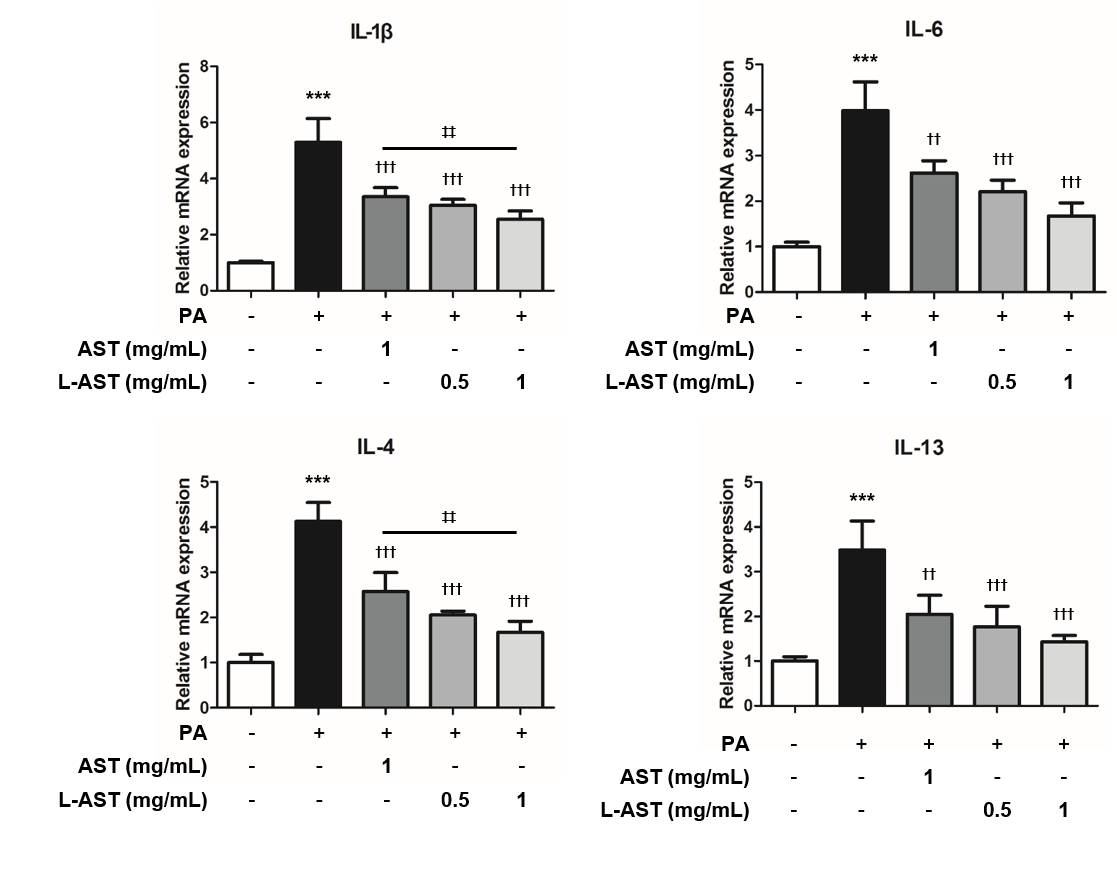


**Supplementary Figure S1. Liposomal astaxanthin reduces inflammation in lymph nodes.** mRNA expression of AD-related cytokines, IL-1β, IL-6, IL-4, and IL-13, in lymph nodes. *n*=4. ^††,‡‡^*p*<0.01 and ^***,†††^*p*<0.001.
